# Persistent Gaps in the Ultimate Mechanisms of Antimicrobial-Induced Bacterial Killing

**DOI:** 10.3390/antibiotics15030244

**Published:** 2026-02-26

**Authors:** Arpita Nath, Alexandro Rodríguez-Rojas

**Affiliations:** 1 Institute of Biology, Freie Universität Berlin, Königin-Luise-Strasse 1-3, 14195 Berlin, Germany; 2 Clinical Department for Small Animals and Horses, Division of Small Animal Internal Medicine, University of Veterinary Medicine, 1210 Vienna, Austria

**Keywords:** antimicrobial effect, killing of bacteria, stress reactions, metabolic state, persister cells, reactive oxygen species (ROS), biofilms, efflux mechanisms

## Abstract

Antibiotics remain pillars of modern medicine, yet the mechanisms underlying bacterial killing remain incompletely understood. This review addresses unresolved questions in antibiotic lethality, focusing on poorly defined cell-level events. How coinciding stress responses combine to drive killing, and how cells prioritise protective pathways are unclear. Metabolic state strongly modulates lethality, as growth rate, nutrient availability, and respiratory activity determine whether damage reaches a fatal threshold. A small subpopulation of genetically identical cells persists through treatment, but the signals governing entry and maintenance of this state remain elusive. The contribution of reactive oxygen species is context-dependent and debated. Species-specific differences in autolysin activation during cell wall targeting lack unifying principles, while ribosome-targeting antibiotics also induce secondary membrane perturbations whose mechanistic links to translation arrest are unresolved. Biofilms further complicate killing by limiting drug penetration and slowing growth, and host factors such as oxygen tension, pH, and immune pressure reshape bacterial responses in ways that are only beginning to be understood. Addressing these blind spots may reveal new vulnerabilities in bacterial physiology and guide the development of therapeutic strategies that improve killing while limiting tolerance and persistence.

## 1. Introduction

Antibiotic resistance is emerging as a global health crisis that threatens many advances of modern medicine. Multidrug-resistant bacteria are spreading rapidly and causing an increasing number of infections that are difficult or impossible to treat, compromising outcomes in surgery, oncology, and critical care [1,2]. Despite this, the development of new antibiotics remains critically weak: few new classes of drugs have been introduced in recent decades, and large pharmaceutical companies have largely withdrawn from antibiotic development due to high costs and low returns [1,2]. This widening gap between resistance and innovation highlights a fundamental limitation: despite their central role in medicine, the molecular mechanisms by which most antibiotics kill bacteria remain incompletely understood. Even well-studied drugs elicit complex cellular responses that are not yet fully resolved.

Understanding antibiotic lethality is essential for improving therapeutic strategies and countering resistance. Defining the molecular pathways of bacterial cell death not only clarifies drug action but also reveals resistance and persistence mechanisms that undermine treatment efficacy [3,4]. Bactericidal antibiotics are frequently associated with oxidative damage, loss of membrane integrity and metabolic instability, yet the contribution and hierarchy of these processes remain debated [5,6]. Without detailed mechanistic insights, antibiotic development is constrained and unintended outcomes such as tolerance or cross-resistance may occur [7]. Investments in basic research on bacterial cell death processes, therefore, offer a crucial avenue to revitalize antibiotic innovation and safeguard antimicrobial efficacy.

A critical matter in antibiotic research is the operational distinction between live, persister, and dead bacterial cells. These states are defined by experimentally measurable features rather than absolute markers. Live cells retain the capacity to resume growth and form colonies under permissive conditions, reflecting preserved membrane integrity. Persister cells are genetically susceptible bacteria that survive antibiotic exposure in a transient, typically growth-arrested state but can reinitiate divisions after antibiotic removal. In contrast, dead cells have irreversibly lost division capacity and fail to recover metabolic activity even when favorable conditions are restored. Importantly, no single physiological parameter such as membrane depolarization or ATP depletion unequivocally defines death. Thus, reversibility of growth arrest remains the most robust criteria distinguishing persistence from irreversible cell death.

Although antibiotics are often classified as bactericidal or bacteriostatic, this distinction is highly context dependent, varying with concentration, exposure time, species, physiological state, and microenvironment. Drugs considered bacteriostatic at therapeutic levels can become bactericidal when cumulative damage exceeds cellular repair capacity [7]. Experimental studies show that canonical bacteriostatic agents, including macrolides, tetracyclines, and chloramphenicol, can cause substantial loss of viability under intensified exposure, yet the mechanistic basis of this transition remains poorly understood [8,9]. The conditions under which bacteriostasis shifts to bactericidal activity, including metabolic state, growth phase, and stress responses, are still underexplored, leaving important gaps in our interpretation of antibiotic action [10]. This lack of clarity limits our ability to predict drug performance and highlights the need for deeper investigation into death pathways triggered by high concentrations of drugs traditionally labelled as bacteriostatic [11].

Antibiotic action in vivo introduces additional complexity, as host environments impose pressures absent from in vitro systems. Phagocytosis, complement activation, antimicrobial peptides, oxidative bursts, and tissue structure can amplify, mask, or reshape drug effects, often producing outcomes that diverge from broth-based predictions [12,13]. Immune cells can either support bacterial clearance by weakening pathogens before antibiotic exposure or, in some cases, provide transient protection by creating intracellular niches with altered drug penetration or reduced oxidative damage [14]. Gradients of oxygen, nutrients, and host-derived factors further impose metabolic heterogeneity that alters susceptibility [15]. Integrating pharmacodynamics with immune biology is therefore essential for understanding antibiotic efficacy during real infections.

Antibiotic activity also varies markedly across bacterial species, complicating treatment outcomes. The same drug can induce rapid killing in one organism while causing only growth arrest in another under similar exposure conditions [16]. These differences arise from variation in cell envelope structure, efflux capacity, permeability, metabolic wiring, and the presence or absence of target-protection systems [17]. Examples include the poor efficacy of β-lactams against organisms with minimal peptidoglycan, the limited activity of many drugs against intracellular pathogens, and the narrow spectrum of agents such as vancomycin, which is effective against Gram-positive bacteria but excluded by the Gram-negative outer membrane [18]. Some antibiotics also shift mode of action across species: chloramphenicol, classically considered bacteriostatic, can be bactericidal in certain Gram-negative or fast-growing organisms when metabolic state and target engagement favour irreversible damage [19]. These species-specific responses make it difficult to generalise antibiotic activity and show why detailed mechanistic work must account for the physiology of each pathogen rather than relying on broad categories [20].

A deeper mechanistic understanding of how antibiotics convert target inhibition into irreversible bacterial death is likely to have important implications for dosing and therapeutic strategy. If lethality depends on specific metabolic states, respiratory activity, or stress thresholds, then drug efficacy may be shaped not only by concentration but also by whether treatment sustains physiological conditions that favor irreversible damage rather than reversible growth arrest. Mechanistic insight may also guide the timing and design of combination therapies, for example, by pairing antibiotics with metabolic potentiators, modulators of redox balance, or inhibitors of protective stress responses that promote tolerance and persistence. In addition, understanding early intracellular accumulation dynamics and killing kinetics could help refine pharmacokinetic and pharmacodynamic targets beyond minimal inhibitory concentration, shifting attention toward exposure profiles that reliably drive cells past recovery thresholds. Together, these considerations underscore how resolving the molecular basis of antibiotic-induced cell death could support more rational dose selection, improved clearance of persistent subpopulations, and ultimately limit the emergence of resistance.

**Figure 1:**
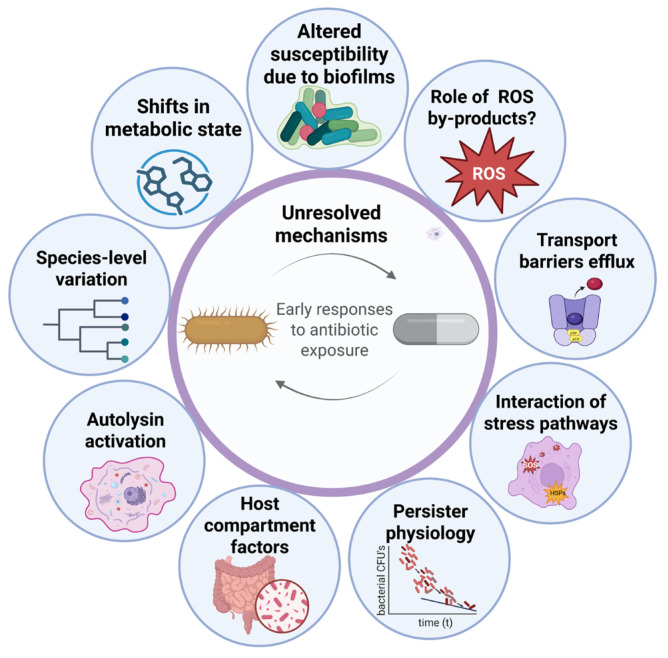
Illustration summarising the key challenges and open questions surrounding how antibiotics ultimately lead to bacterial death. The figure highlights major hypotheses, unresolved mechanisms, and potential directions for future investigation discussed in this review (partially created with biorender.com).

A clearer view of these challenges (see Figure 1) is needed. This review synthesises key unresolved questions surrounding antibiotic-induced bacterial death and outlines directions for future work, including how multiple stresses interact within cells, how metabolic flux determines irreversibility thresholds, and why closely related species respond so differently despite sharing targets. Addressing these gaps may reveal new vulnerabilities, clarify host contributions to clearance, and support the development of combination therapies, potentiators, and treatment strategies that limit tolerance and persistence (Table 1).

Species-level variation is one of the most striking yet least understood features of antimicrobial action. Classical pharmacodynamic studies already demonstrated that minimal inhibitory concentration fails to predict killing dynamics: species with similar MICs can differ substantially in lag times and death rates [21]. While concepts such as resistance, tolerance, and persistence capture this diversity, they do not explain why identical molecular exposures lead to death in some organisms but only growth arrest in others [22]. Time-kill studies with clindamycin illustrate this problem, revealing species-specific differences in ribosome binding and killing kinetics despite equivalent susceptibility classifications [23]. Why a given pattern of target occupancy and translational arrest translates into fast killing in one organism but into mere growth arrest in another lingers largely unsettled. Work on chloramphenicol illustrates this gap especially well. Textbooks still label it as bacteriostatic, even though clinical and experimental data indicate bactericidal behaviour against some pathogens, particularly meningitis isolates, while it is mainly static against enteric Gram-negative bacilli [24,25]. Emerging evidence suggests that species-specific stress and stringent response pathways influence whether cells enter protective low-activity states or progress toward lethal damage, but the sequence linking target inhibition to death remains incompletely mapped [26,27].

Similar uncertainties apply across drug classes. Differences in permeability, porins, efflux systems, and envelope stress responses modulate intracellular drug levels, yet the rate-limiting steps for killing are rarely defined for individual species. Variability in metabolism, redox balance, and respiratory architecture further shapes damage accumulation, but quantitative links to killing kinetics remain fragmentary [28,29]. Community-level studies show that antibiotic effects also depend on microbial context, as neighbouring species can buffer pH, detoxify reactive compounds, or alter nutrient availability [30,31,32]. Together, these findings highlight the limits of generalising from a few model organisms and emphasise the need for a systematic, species-spanning framework that traces the molecular sequence from drug binding to irreversible loss of viability.

## 2. Metabolic State Affects Cell Death Efficacy

Metabolic state is considered an important predictor of how efficiently antibiotics kill bacteria, but the key causal links are still far from clear. Growth rate alone does not explain lethality: cells with similar doubling times can display very different killing curves under identical exposure, indicating that deeper features of central metabolism and respiration shape outcomes [33]. Some studies have linked bactericidal activity to active respiration, showing that many killing antibiotics drive an increase in respiratory flux, whereas bacteriostatic agents tend to dampen it [34]. When respiration is chemically or genetically reduced, killing often collapses; when respiration is boosted, lethality can be restored [35]. Nevertheless, it is unknown which parts of the respiratory chain are truly required for death in each context, which redox changes are causal rather than secondary, and how these relationships vary across species and drug classes. Single-cell measurements reveal strong heterogeneity in metabolic activity within isogenic populations, but there is still no general rule that predicts how variation in ATP levels, membrane potential, or redox state shifts a given cell from survival to death during treatment [36].

Nutrient content adds another conflicting layer. Switching carbon sources, altering amino acid availability, or modifying micronutrient levels can transform a tolerant population into a susceptible one and vice versa [37,38]. Supplementing specific metabolites can activate slow-growing cells and make them easier to kill, but the spectrum of possible nutrient drug combinations is vast, and only a fraction has been examined systematically [39]. Even within individual pathways, such as purine metabolism, the literature is inconclusive: some studies report that high purine availability reduces efficacy, while others show that purine depletion can also promote tolerance, suggesting a non-linear response that current frameworks cannot capture [40]. Global metabolomics and flux analyses show extensive rewiring after antibiotic exposure, including changes in fermentation, the tricarboxylic acid cycle, amino acid catabolism, and cofactor balance [34,41]. However, most of these shifts are described as broad signatures rather than connected to defined steps on the route to death or survival. We often observe that a metabolite pool changes but cannot tell if the meaning of this shift is protective, harmful, or simply a consequence of stress. The quick transition of cells between metabolic states during antibiotic exposure, how spatial gradients in tissues or biofilms shape these transitions, or how host-derived factors influence respiration and nutrient use in vivo also remain cryptic. Addressing these blind spots will require experiments that track metabolic flux, respiratory activity, and lethal damage in parallel, ideally at single-cell resolution and across diverse physiological conditions, to separate true causal events from secondary stress responses.

## 3. Mechanisms Underlying Persister Survival Despite Genetic Identity

One of the most enduring puzzles in antibiotic biology is why a small fraction of genetically identical cells routinely survives treatment while the majority dies. These survivors, known as persisters, are not mutants and do not carry heritable resistance traits. Instead, they exist in a transient physiological state that allows them to tolerate concentrations of antibiotics that are lethal to their siblings. Although the persistence phenomenon was described more than seventy years ago, the precise molecular steps that initiate, stabilise, and terminate this state remain largely unknown [42]. Much effort has gone into identifying the cellular features that distinguish persisters from non-survivors; however, the mechanistic chain that leads a particular cell into this state is still highly fragmented and incomplete.

A central unanswered question concerns the origin of persistent heterogeneity in otherwise uniform populations. Early models proposed that persisters arise from rare, slow-growing variants that exist before antibiotic exposure. However, single-cell microscopy studies have demonstrated that some persisters do not start in an obviously slow state and that antibiotic exposure can itself drive cells into persistence through mechanisms independent of growth rate [43]. This raises questions about what the relevant triggers are and how rapidly a cell can transition into a protected physiological state. Although toxin-antitoxin modules were once considered primary drivers of persistence, deletion of many such modules does not abolish the phenotype, indicating that these systems are at best contributors and not universal switches [44]. The question is whether these modules act as stress amplifiers, stochastic noise generators, or downstream effectors is still unresolved.

Energy depletion has also been suggested as a key determinant, with studies reporting that persisters are often characterised by low ATP content. Regardless of this observation, we ignore whether ATP depletion causes persistence or instead occurs as a late consequence of shifting into a protected state [45]. Similarly, alterations in membrane potential, proton motive force (PMF), and redox balance have been linked to survival, but the field lacks models that incorporate these features into a coherent mechanistic framework. For example, some data suggest that a collapse in respiratory activity protects cells by reducing antibiotic uptake or target engagement, but other studies show that respiration plays different roles depending on species, antibiotic class, and nutrient conditions [46]. Without real-time, single-cell measurements that track these processes in parallel, it is not possible to separate causal events from secondary stress signatures.

Another largely debatable topic concerns the diversity of persister types. It is now clear that not all persisters share the same physiological state. Some arise from slow-growing cells, others from metabolically active cells that undergo rapid shifts in behaviour, and still others may form through oxidative stress responses or envelope stress pathways [47]. Whether these different routes converge on a final common protected state or represent fundamentally distinct phenotypes is unknown. Furthermore, the possibility that antibiotics trigger specific persister states depending on their mode of action has not been rigorously tested. Aminoglycosides, β-lactams, and fluoroquinolones target different cellular processes, so the survival pathways they select for may also vary. The field lacks a comprehensive classification of persister subtypes across species and antibiotics, which limits the ability to generalise findings.

Species-specific differences further complicate the picture. Most mechanistic insights have been derived from a small number of model organisms such as *Escherichia coli* and *Staphylococcus aureus*. Yet environmental bacteria, intracellular pathogens, opportunistic pathogens, and slow-growing species may enter persistence through distinct routes. For example, Mycobacterium species display marked persistence against multiple drugs, but the relevant physiological traits appear to differ from those of Gram-negative enteric bacteria [48]. How conserved or divergent the core persistence mechanisms are across species remains unknown. This lack of comparative work restricts the clinical relevance of existing models.

The exit from the persistent state continues to be not well understood. While some studies show that persisters quickly resume growth once antibiotics are removed, others suggest that exit is gradual and may involve reactivation of metabolic pathways, restoration of PMF, or reorganization of chromosomal domains [49]. Whether exit is a passive return to homeostasis or an active, regulated process is still not well explained. Understanding this step is essential, as the timing of exit influences relapse, treatment duration, and evolution of resistance.

## 4. Multiple Stress Responses Shape Cellular Fate Under Antibiotic Exposure

Antibiotic exposure rarely activates a single stress response. Most antibacterial agents provoke several injury signals at the same time, including DNA damage, ribosome stalling, membrane disruption, oxidative shifts, redox imbalance, and metabolic defects. Although each pathway has been analysed separately, its interactions within single cells are one of the least characterised aspects of bacterial killing. The field still lacks a model that explains how antibiotics convert complex stress signatures into irreversible death, and which combinations of stresses are most predictive of lethality [50].

A major unresolved question is how cells prioritise protective circuits when several responses are activated simultaneously. A single bacterium may trigger the SOS network to repair DNA lesions, turn on envelope maintenance pathways to counteract membrane perturbation, and activate heat shock regulons to manage misfolded proteins. These systems should, in principle, cooperate to maintain viability, but experimental work shows that they often interfere with one another. Activation of the SOS response can slow growth and indirectly weaken envelope integrity, while envelope stress pathways can influence ribosome assembly and affect global translation [37]. How cells integrate or fail to integrate these overlapping signals is still not clear, and we still do not know how certain regulons dominate antibiotic contexts or if conflicts between them determine survival.

Another open issue concerns the extent to which antibiotic-induced stress responses reflect lethal damage rather than compensate for it. Many stress indicators used in research are correlational. Oxidative stress gene upregulation, efflux activation, and protein quality control responses frequently rise during exposure, but at this point, it remains uncertain which responses participate directly in killing and which simply accompany injury [51]. Bactericidal antibiotics often increase reactive oxygen signals, but the contribution of these species to death is still debated, with some studies reporting ROS-dependent killing and others observing efficient killing with little ROS involvement [51]. Without real-time single-cell measurements, causality cannot be confidently assigned.

Protein stress contributes to further uncertainty. Antibiotics that disrupt transcription or translation generate incomplete or misfolded peptides that overwhelm proteostasis systems. Chaperones and proteases attempt to remove these products, but it is still poorly defined if the collapse of proteostasis is itself a lethal event or if there is an interaction with membrane damage, DNA breaks, or metabolic stress to push cells beyond the point of recovery. Evidence suggests that misfolded proteins can trap or disable key repair proteins, leading to secondary failures in envelope maintenance and division, but the thresholds at which protein stress becomes lethal are not well defined [52].

Envelope-linked processes also intersect unpredictably with other stresses. Cell wall targeting antibiotics activate autolysins, which can accelerate membrane damage and lysis. However, the timing and magnitude of autolysin activity vary widely between species and environmental conditions, and the influence of oxidative or translational stress on these processes is not known. There is some evidence that connections exist between envelope stress systems and the stringent response, but the details of this interplay are incomplete [53]. Without clarity on these links, it is difficult to predict when envelope stress will transition from repairable injury to irreversible damage.

Although many stress signatures correlate with strong killing, their predictive value is weak. Transcriptomic and proteomic profiles often show extensive changes after antibiotic exposure, but only a fraction of these alterations relate directly to lethality. Similar stress patterns can be observed in cells that recover after treatment and in those that die. As a result, current stress markers cannot reliably predict killing efficiency, especially when comparing different species or growth conditions. These unresolved areas limit efforts to design adjuvant therapies aimed at intensifying lethal stresses or weakening protective ones.

## 5. Reactive Oxygen Species as Modulators of Cell Death

Reactive oxygen species (ROS) have been proposed as shared contributors to killing by many bactericidal antibiotics, yet this idea remains a controversial point in the field. Early work suggested that diverse drug classes, including β-lactams, aminoglycosides, and fluoroquinolones, converge on a common oxidative cascade that generates hydroxyl radicals and secondary damage beyond the primary drug target [54]. This model helped explain why different antibiotics can show similar late killing phenotypes and why metabolic state influences lethality. However, subsequent studies failed to reproduce key findings or reached opposite conclusions, leading to a long-running debate about the role of ROS as a central mediator of death, context-dependent amplifiers, or mostly as byproducts of antibiotic stress [55,56].

Contradictions begin with basic detection. Several groups reported increased ROS indicators, such as fluorescent dyes or oxidative damage markers, during exposure to bactericidal antibiotics, and showed that ROS scavengers or anaerobic conditions reduce killing [57,58]. Nevertheless, other studies found that commonly used ROS probes are nonspecific, that scavengers can have off-target effects, and that some antibiotics retain full activity in the apparent absence of measurable ROS. In some cases, conditions that strongly increased ROS readouts did not enhance killing, while in other cases, efficient killing occurred with little change in standard ROS markers. These discrepancies raise questions about how reliably ROS signals report the processes that determine cell fate [53,59].

A second area of disagreement concerns genetic evidence. Mutants defective in respiratory chain components, iron homeostasis, or oxidative stress responses sometimes show altered sensitivity to bactericidal drugs, which has been interpreted as support for an ROS-dependent component of killing [60,61]. However, many of these mutations also change basal metabolism, membrane potential, or drug uptake, making it difficult to separate specific ROS effects from broader physiological shifts. Conversely, deletion of classical antioxidant defences such as catalases, peroxidases, or superoxide dismutase often has modest or no impact on antibiotic killing under standard conditions [62,63]. One would expect a dominant ROS-based mechanism to produce a stronger and more consistent signature in these mutants; however, the literature is mixed and sometimes contradictory, even within the same species and antibiotic class.

There is also a striking lack of systematic work using overexpression of ROS scavenging systems to test causal roles in an unbiased way. Many studies have relied on deletion mutants or chemical scavengers, both of which can introduce confounding effects on growth, respiration, or redox balance [64,65]. In contrast, experiments that robustly overproduce catalases, peroxidases, or superoxide dismutase from strong promoters and then measure time-kill curves under well-controlled conditions are rare. Such designs would offer a more direct test: if ROS makes a decisive contribution to death for a given drug and species, then strongly enhanced detoxification capacity should reproducibly delay or attenuate killing. The shortage of these gain-of-function studies leaves a key question open: to what extent can bolstering antioxidant systems truly protect cells from otherwise lethal antibiotic exposure across conditions and bacterial backgrounds?

Differences in drug class and species further complicated the interpretation. For aminoglycosides, some data support a model in which mistranslation leads to misfolded proteins, membrane damage, and respiratory stimulation that generates toxic ROS, while other results show effective killing in mutants or conditions where ROS appear minimal [66,67]. For fluoroquinolones, there is clearer evidence for primary lethal lesions in DNA gyrase and replication forks, but the extent to which secondary oxidative damage accelerates or propagates killing is under dispute [68,69]. β-lactams present another pattern, where autolysin-driven lysis is a dominant feature, but studies disagree on how ROS modulate this process or simply co-occur as part of a disturbed metabolic state [12,70]. Systematic, side-by-side comparisons of ROS involvement across several antibiotics in the same strain, and across several strains for the same antibiotic, are still scarce.

Environmental context adds another source of uncertainty. Oxygen availability, iron levels, nutrient status, and growth phase can all alter both ROS formation and antibiotic activity. Some of the most robust ROS effects are reported in rich media and rapidly growing cells, whereas pathogens in host tissues often inhabit oxygen-limited, nutrient-constrained, or biofilm-associated environments [71,72]. Under those conditions, the relevance of ROS-mediated damage may change dramatically, but in vivo and biofilm studies directly quantifying ROS contributions to killing remain limited. This shortfall in the ROS–antibiotic debate, derived from studies using planktonic, well-aerated cultures, may be relevant to clinical settings or natural environments such as soil, where antibiotics diffuse slowly.

## 6. Autolysin Activation as a Consequence of Cell Wall Targeting Antibiotics

Cell wall-active antibiotics disrupt peptidoglycan synthesis; nonetheless, the way these drugs trigger autolysins, the endogenous enzymes that cleave the cell wall, is still one of the most debated aspects of bacterial killing. Autolysins normally function in controlled remodelling during growth and division, but under antibiotic stress, they can cause catastrophic wall breakdown and rapid lysis. Despite decades of study, the precise molecular steps that activate autolysins and why these steps differ so markedly across bacterial species are incompletely described [73].

The classical model proposes that β-lactams inhibit penicillin-binding proteins (PBPs), which disturbs the balance between cell wall synthesis and turnover. This imbalance is theorised to expose nascent peptidoglycan or uncrosslinked strands that stimulate autolysin activity. However, this view is too simplistic for many organisms. Some bacteria lyse readily after β-lactam exposure, while others maintain shape or die without visible lysis, even with similar degrees of PBP inhibition [73,74]. These inconsistencies suggest that autolysin activation is not an automatic consequence of blocked synthesis but depends on additional regulatory layers that vary between species.

One major unknown concern is how envelope stress responses modulate autolysin activity. In *Bacillus subtilis*, the sigma M and sigma W regulons influence expression and activity of several autolysins, but deleting these pathways does not fully eliminate lysis after β-lactam treatment, suggesting redundant or compensatory controls [75]. In contrast, *Staphylococcus aureus* shows strong dependence on the WalKR two-component system, which regulates major autolysins such as Atl. Even in *S. aureus*, the timing of autolysin activation differs depending on the specific β-lactam, growth phase, and metabolic state, indicating context-dependent wiring that we currently do not understand [76].

Conflicting results also exist regarding the role of teichoic acids. Wall teichoic acids are thought to inhibit autolysins by restricting access to peptidoglycan. Some studies report that inhibiting teichoic acid synthesis increases autolysin activity and sensitises cells to β-lactams, while others find minimal effects or strain-specific returns [51]. These contradictory findings suggest that teichoic acid regulation of autolysin activity is highly dependent on species, growth conditions, and possibly the biochemical properties of individual autolysins.

Another unresolved area is the behaviour of Gram-negative bacteria. Because Gram negatives possess an outer membrane and a thinner peptidoglycan layer, their lysis patterns differ greatly from Gram positives. Some species undergo rapid lysis when PBPs are inhibited, whereas others resist lysis and instead form spherical cell wall-deficient shapes before death [36]. The reasons behind this divergence remain confusing. It is not known whether autolysins are less responsive, or the outer membrane provides mechanical stabilization that delays lysis, or if species differ in how they coordinate peptidoglycan synthesis with hydrolysis.

Even the identity of the key autolysins involved is uncertain for many organisms. Several bacteria encode dozens of peptidoglycan hydrolases, and knocking out individual enzymes often produces mild phenotypes due to redundancy. This redundancy complicates efforts to map the cascade leading to lethal wall cleavage. Moreover, very few studies track autolysin activity in real time during antibiotic exposure, so the temporal order of events leading to lysis is largely unknown [52].

The environmental and physiological context appears to strongly influence autolysin behaviour. The same antibiotic can trigger rapid lysis in fast-growing cells but minimal lysis in nutrient-limited or stationary phase populations. Biofilm-grown cells often resist lysis despite exposure to drugs that readily lyse planktonic cells [77,78]. How metabolism, growth rate, and osmotic conditions influence autolysin activation have not been explored.

## 7. Consequences of Ribosome Inhibition

Ribosome-acting antibiotics are often framed as simple blockers of protein synthesis, but growing evidence shows that many members of this group provoke downstream disturbances that reach well beyond the ribosome. These secondary responses include membrane depolarization, altered permeability, oxidative shifts, metabolic imbalance, and collapse of protein quality control. Although these effects are repeatedly documented, their actual contribution to killing remains debated, and the mechanistic path linking ribosomal inhibition to later stages of cellular damage is still not settled [79,80,81].

A persistent question concerns the origins of membrane damage triggered by ribosomal inhibitors. One explanation, supported by several studies, is that drugs induce strong misreading, particularly aminoglycosides, leading to the production of faulty membrane proteins that fail to fold or insert correctly. When these aberrant proteins incorporate into the bilayer, they can create pores or destabilise the electrochemical gradient, providing a plausible mechanistic route from ribosomal miscoding to lethal membrane failure [82]. However, other works report membrane depolarization even when mistranslation is chemically suppressed, suggesting that mistranslation might be neither necessary nor sufficient for membrane injury. These conflicting datasets have prevented a consensus about the role of membrane damage as a direct by-product of miscoding, a downstream effect of broader physiological stress, or a combination of both.

The situation becomes even more complex when comparing how different ribosome-targeted drugs influence secondary injury. Aminoglycosides commonly produce rapid permeability changes, whereas tetracyclines suppress translation without immediate envelope disruption. Even within aminoglycosides, the speed and magnitude of membrane damage vary by species and growth state, indicating that physiological wiring strongly shapes the downstream consequences of ribosomal inhibition [82,83]. This variability has implications for understanding why most aminoglycosides are bactericidal while spectinomycin, despite also binding the 30S subunit, is largely bacteriostatic. Classic aminoglycosides such as gentamicin or kanamycin bind the 30S decoding centre and induce substantial misreading, generating a pool of mistranslated, membrane-destabilizing proteins. Some members of this class also contact regions of the 50S subunit, which may alter the pattern of stalling or faulty proteins generated and further accelerate envelope damage. In contrast, spectinomycin binds a distinct site on the 30S subunit, blocks translocation, and causes translational arrest with minimal miscoding. Because fewer defective membrane proteins arise, envelope integrity is far more stable. This difference is the leading hypothesis for the divergent bactericidal versus bacteriostatic mode of action, yet it still does not explain species-specific exceptions or why spectinomycin can occasionally cause a harsher impact under certain conditions.

Metabolic factors, which greatly influence bacterial physiology, account for differential killing susceptibility. Some ribosomal inhibitors reduce ATP consumption and slow respiration, while others increase respiratory flux or generate altered redox states. Because shifts in respiration influence membrane potential, PMF, and reactive species production, it becomes difficult to know where the depolarization comes from: mistranslation, metabolic imbalance, or direct drug–membrane interactions [55,84]. Importantly, membrane depolarization does not necessarily equate to a complete loss of the proton motive force, as compensatory adjustments in the transmembrane pH gradient can preserve overall bioenergetic homeostasis under physiological conditions. These opposing metabolic effects complicate attempts to link specific downstream changes to killing and highlight how environment-dependent these cascades are.

Another issue involves envelope maintenance pathways. Reduced expression of certain membrane or cell wall proteins during translation inhibition could weaken integrity over time, but evidence is mixed. Some reports show decreased levels of essential envelope factors, while others find that cells preferentially preserve the synthesis of these proteins despite strong ribosomal inhibition. If selective retention does occur, then ribosome inhibitors may not simply shut down protein synthesis uniformly but instead create imbalances that indirectly destabilise the envelope [85]. Time-resolved proteomics combined with physiological measurements is still lacking to resolve this point.

Species-specific differences further complicate interpretation of research results. Gram-positive bacteria often display stronger membrane depolarization than Gram-negative organisms when exposed to comparable ribosome inhibitors, whereas mycobacteria show marked oxidative stress with relatively stable membranes during early exposure [37]. The mechanistic basis for these divergent patterns is uncertain and may involve variations in envelope composition, ribosomal protection proteins, or stress-response networks.

Importantly, it is still opaque whether secondary stresses, membrane injury, oxidative surges, and metabolic shifts are core components of lethal action or merely indicators of severe physiological decline. In many settings, these downstream signatures correlate with killing, but examples exist where translation inhibition halts growth without detectable envelope damage, and others where membrane disruption does not translate into rapid death. Current evidence, therefore, cannot distinguish causation from correlation of these effects and their contribution to the collapsing cellular organization [86].

## 8. Spatial Structure Governs Antibiotic Response in Biofilms

Biofilms profoundly modify how antibiotics act during the first minutes and hours of exposure. Rather than behaving as simple diffusion barriers, they create highly uneven microenvironments that reshape drug penetration, local physiology, and the timing of killing. Even when the bulk matrix offers only modest physical resistance, gradients of oxygen, nutrients, pH, and redox state arise within tens of micrometres, producing cell subpopulations with distinct metabolic states. These differences strongly influence whether an antibiotic triggers fast death, delayed injury, or little short-term effect at all [57,87].

A major unresolved issue is the relationship between matrix composition and immediate drug activity. Some studies report that polysaccharides, DNA, or amyloid fibres hinder the transit of positively charged drugs, whereas others find that many agents diffuse through the matrix almost unimpeded [59]. The contradiction suggests that impeded penetration alone rarely explains the reduced killing observed during early exposure. Instead, low metabolic activity in deeper layers, combined with altered proton-motive force or reduced uptake, may blunt the first wave of antibiotic action. However, the degree to which these physiological states are stable or reversible in the early phase of treatment is still uncertain, and different species form matrices with very different chemical properties.

Another area that stays improperly defined is the formation of protective spatial niches. Cells in biofilms do not experience antibiotics uniformly: drug concentrations, oxidative conditions, and membrane potential vary sharply with depth. This results in heterogeneous killing, with surface layers often dying rapidly while inner regions stay intact for hours. Whether this pattern is driven mainly by differential uptake, local stress responses, or structural shielding is unclear. Live-imaging and microfluidic studies show that even small changes in flow conditions or matrix density can switch a drug from rapidly bactericidal to nearly inactive during the early phase [57,87].

Interactions between antimicrobial classes and the matrix add further complexity. Agents that rely on active uptake or strong respiration often show severely reduced early activity in anoxic or slow-growing zones, even when concentrations are high. By contrast, drugs targeting cell wall synthesis may reach their targets more uniformly but still display uneven activity because only actively growing sectors of the biofilm are susceptible [57,87]. These patterns vary widely across species and strains, highlighting how difficult it is to predict immediate drug impact from planktonic behaviour alone.

A crucial knowledge deficit arises from early injury signals in biofilms, such as small permeability changes or local oxidative bursts, and whether these factors meaningfully contribute to long-term survival or is simply a transient disturbance. Some studies link early membrane damage at the periphery to later detachment or collapse, whereas others find no connection between initial injury and eventual killing [58,88]. Without high-resolution time-series data, the field cannot determine whether the early phase is mechanistically decisive or merely sets the stage for slower, physiology-based survival.

## 9. Efflux System Dynamics During Initial Phases of Antibiotic Treatment

The earliest phase of antibiotic exposure is shaped strongly by how bacteria control the entry and exit of drugs. Efflux pumps, porin channels, and membrane permeability barriers act within seconds to minutes, often determining whether intracellular concentrations ever reach inhibitory or lethal levels. Although efflux is often discussed in the context of long-term resistance, many pumps operate at high basal activity during the first contact with antibiotics, suggesting that they play an immediate role in shaping early pharmacodynamics [89,90].

One major unresolved issue is the relative importance of constitutive pump activity versus induced responses. Several multidrug transporters, such as AcrAB-TolC in Gram-negative bacteria, expel a broad range of agents at rates sufficient to counter incoming flux [91,92]. This suggests that the first minutes of exposure may already reflect a balance between passive diffusion through porins and active removal through efflux. Nevertheless, other studies find that some transporters require transcriptional activation or substrate-triggered conformational shifts before reaching maximum capacity. It is still not known if this induction is fast enough to shape the initial phase of intracellular accumulation, and the dynamics likely differ among species.

Porin-mediated entry represents another critical variable. Many antibiotics cross the outer membrane mainly through general diffusion pores such as OmpF or OmpC, but expression levels of these channels fluctuate with environmental conditions. Even small reductions in porin abundance can slow uptake enough to delay or blunt the initial intracellular rise in drug concentration [93]. However, some drugs diffuse through the outer membrane independently of major porins, and in these cases, the influence of transport channels on early accumulation is limited [93,94]. Contradictory findings across species and growth conditions have made it difficult to generalise how porin composition shapes the earliest phase of exposure.

The extent to which active efflux shapes short-term survival is also under debate. Strong efflux activity can prevent intracellular levels from ever reaching thresholds required for rapid killing, but in other cases, efflux appears insufficient to counter high extracellular concentrations during the first minutes. Some studies show that efflux-deficient mutants accumulate antibiotics within seconds, resulting in rapid depolarization or growth arrest, whereas others report little difference between wild-type and efflux-deficient strains in the early phase [95].

Transport barriers also operate at the level of membrane PMF. Drugs that depend on the proton motive force for uptake, such as aminoglycosides, show immediate changes in entry rates when membrane potential fluctuates. Even minor reductions in membrane potential can slow uptake substantially, giving cells a brief window to activate protective systems before lethal concentrations accumulate [65,83]. However, whether such changes occur quickly enough to influence the earliest events of drug action is undetermined, and reported data vary widely between species.

Spatial heterogeneity further complicates the picture. Even in planktonic cultures, subpopulations with lower membrane potential or altered porin profiles may accumulate drugs slowly, creating early tolerance pockets that survive the initial exposure [96]. These subpopulations can seed surviving lineages that later drive persistent or recurrent infection. Only a few studies have mapped transport variability at high temporal resolution [97], leaving a major void in understanding how it shapes early killing.

In summary, while efflux systems and transport barriers clearly influence antibiotic action, the extent to which they shape the very first minutes of exposure remains contested. Differences in pump activity, porin expression, membrane PMF, and population heterogeneity make it difficult to determine which mechanisms dominate at early times.

## 10. Host Physiological Conditions Shape Antibiotic Lethality

Antibiotic activity inside host tissues rarely reflects what is measured in standard laboratory media. Local conditions such as pH, immune competence, and oxygen availability can alter uptake, target engagement, and downstream physiological responses in ways that are often overlooked. These host-driven variables shape the first minutes and hours of exposure, whether drugs achieve effective concentrations or trigger the expected cellular injuries [72,77,98].

The pH is one of the most influential but poorly predicted modifiers. Acidic environments, common in abscesses, infected wounds, and certain intracellular compartments, can reduce the uptake of weakly basic antibiotics and slow their diffusion through host-derived exudates [99,100]. For aminoglycosides and macrolides, reduced pH weakens membrane potential, diminishing entry and delaying the onset of killing. Conversely, some β-lactams keep the activity under acidic conditions, although reports conflict on how much activity is preserved. The contradictory data reflect substantial variation between tissue sites and between species, highlighting the need for more precise measurements of in situ drug patterns of action.

Immunosuppression of patients influences the effectiveness of antimicrobials in vivo. Many antibiotics rely on cooperation with innate immunity to clear infections efficiently. Neutrophils and macrophages can damage bacterial envelopes, release antimicrobial peptides, and create oxidative bursts that enhance the action of several drug classes. In hosts with reduced immune activity, these synergistic effects are lost, often resulting in partial or delayed killing even when drug levels are adequate [101]. However, the exact contribution of each immune component continues to be only partially characterised, and data from animal models often conflict with clinical observations. Some studies show that immunosuppressed hosts experience higher levels of persisters after antibiotic treatment, whereas others find no change. The lack of agreement has made it difficult to determine which immune functions are essential for early enhancement of antibiotic action.

Oxygen partial pressure also strongly influences killing, particularly for drugs that depend on aerobic metabolism or membrane potential for uptake. Low-oxygen sites such as necrotic tissue, granulomas, or limited perfused lesions often contain slow-growing or hypoxic cells that import drugs more slowly. This is especially relevant for aminoglycosides and fluoroquinolones, which require active respiration for efficient entry. Under hypoxia, uptake slows, intracellular concentrations do not build up, and downstream lethal events such as membrane depolarization or DNA damage may be blunted [102,103]. However, some reports indicate that certain antibiotics generate modest activity even under low oxygen, suggesting alternative uptake routes or partial preservation of metabolic activity. These observations are difficult to reconcile and point to substantial shortcomings in understanding how oxygen availability shapes the early phase of killing.

Host-derived stresses can interact unpredictably. Acidic pH combined with hypoxia can lead to extremely low membrane potential, which affects both uptake and efflux. Immune factors can either enhance or hinder antibiotic penetration depending on the extent of inflammation, tissue swelling, or release of reactive species. Immune effectors like antimicrobial peptides can induce persistence and tolerance in bacteria [104]. Such cross-effects are rarely examined in combination, leaving uncertainty about how in vivo conditions modulate early drug action.

Despite the recognised influence of these factors, most pharmacodynamic models still rely on data generated under ideal laboratory conditions. As a result, predictions of killing in living tissue often fail to match clinical efficacy. A better mechanistic understanding of how pH, immune activity, and oxygen tension shape early antibiotic action will require integrated studies combining tissue-level measurements, single-cell tracking, and in situ pharmacokinetics [105,106].

## 11. Structural Biology Perspectives on Antibiotic Killing Mechanisms

Antibiotic killing mechanisms are classically defined by the inhibition of essential bacterial processes, yet from a structural biology perspective many aspects of how these interactions translate into cell death remain incompletely understood [36,107]. During decades, structural work has resolved atomic level interactions between antibiotics and their targets, these static views often fail to capture the dynamic and kinetic features that ultimately determine bactericidal versus bacteriostatic outcomes [108]. A central unresolved question is how binding events at individual targets propagate into irreversible physiological collapse, particularly under heterogeneous cellular and environmental conditions.

β-lactam antibiotics and their primary targets, penicillin binding proteins (PBPs), represent one of the most structurally characterised antibiotic systems. Crystal structures and cryo-electron microscopy studies have elucidated how β-lactam acylates the active site serine of PBPs, thereby inhibiting transpeptidation during peptidoglycan synthesis [109,110]. However, key gaps remain regarding target selectivity among multiple PBPs, the structural basis of differential killing rates, and the contribution of binding kinetics to lethality rather than simple growth inhibition [111]. Furthermore, the spatial organization of PBPs within the membrane, their dynamic interactions with other cell wall enzymes, and the coupling of local enzymatic inhibition to global envelope failure remain poorly resolved from a structural standpoint [112].

Ribosome targeting antibiotics provide another area where structural information is extensive but mechanistic understanding is incomplete. High-resolution structures have shown how tetracyclines bind to the A site of the 30S ribosomal subunit to block tRNA accommodation, how aminoglycosides induce conformational distortions in the decoding centre that promote mistranslation, and how chloramphenicol inhibits peptidyl transferase activity within the 50S subunit [113,114,115]. Macrolides bind within the nascent peptide exit tunnel, where they selectively interfere with elongation in a context-dependent manner [9]. Despite these insights, structural biology has yet to fully explain how ribosomal inhibition leads to bacterial killing, particularly for aminoglycosides, where mistranslation, membrane damage, and metabolic stress appear to be mechanistically linked [116].

A major unresolved area concerns membrane transporters and intracellular antibiotic accumulation. For many antibiotic classes, including tetracyclines, aminoglycosides, and macrolides, uptake depends on limited characterised transport pathways or membrane potential driven processes [117]. Efflux pumps, which are central to intrinsic and acquired resistance, are structurally diverse and highly dynamic. Although individual transporter structures have been solved, there is limited structural insight into antibiotic binding kinetics, substrate competition, and transporter behaviour within native membrane environments [118]. This lack of information constrains our ability to predict intracellular drug concentrations and target occupancy, both of which critically influence killing efficiency.

Recent advances in machine learning, particularly AlphaFold developed by DeepMind, have begun to address some of these limitations by enabling high confidence structural models of antibiotic targets, resistance enzymes, and membrane proteins [119]. AlphaFold predictions have supported the interpretation of resistance mutations in PBPs and ribosomal proteins, enabled modeling of transporter architectures, and facilitated hypotheses regarding protein interactions within essential bacterial complexes [120]. However, these models largely remain static and do not yet capture binding kinetics, conformational transitions, or antibiotic induced remodeling of macromolecular assemblies.

## 12. Concluding Remarks

Despite decades of research, key mechanistic steps linking antibiotic exposure to bacterial cell death remain unresolved. Many drug classes trigger multiple stress responses at once, making it difficult to identify which events are mechanistically central and which are secondary disturbances. Key uncertainties include the extent to which mistranslation contributes to membrane failure during aminoglycoside treatment, why similar ribosomal lesions lead to bacteriostasis in some cases and rapid killing in others, and how metabolic state shapes these outcomes. The long-standing debate about the contribution of ROS reflects the broader challenge of separating causes from correlation in complex stress networks.

Biofilm-associated cells add further complexity, as spatial gradients in metabolism, oxygen, and membrane potential produce heterogeneous responses within minutes of exposure. Efflux systems and transport barriers can modulate early intracellular drug levels, but their influence varies strongly between species and physiological states. In vivo, host factors such as pH, oxygen tension, and the level of immune activity have a role on the downstream cascade of injury that follows. These variables help explain why the lethal effects observed in rich laboratory media often fail to predict killing in tissues.

A complete understanding of antibiotic lethality will require approaches that integrate single-cell physiology, real-time metabolic measurements, structural analyses, and in situ pharmacokinetics. Only by resolving the early events with higher temporal and spatial resolution will it become possible to build predictive models that explain why some cells die within minutes, others survive despite identical genomes, and how therapeutic conditions modify these trajectories.

This review highlights that antibiotic lethality cannot be fully understood by focusing on drug targets in isolation. Killing efficiency instead emerges from interactions between metabolic, physiological and spatial state of the bacterial cell at the time of exposure. Growth rate alone is a poor predictor, as cells with similar division rates can show markedly different killing dynamics depending on respiration, redox balance, and energy status. As a result, discovery pipelines that rely primarily on growth inhibition or minimal inhibitory concentration risk overlooking compounds whose efficacy depends on metabolic context. Identifying pathogen and environment specific metabolic configurations that render cells vulnerable to irreversible injury may therefore offer a more reliable path forward. Single-cell heterogeneity further reinforces this need, as tolerance and persistence often arise from transient physiological states rather than fixed genetic differences.

Nutrient availability adds another layer of complexity that is rarely incorporated into antibiotic development. Shifts in nutrients can strongly alter susceptibility, creating opportunities for rational drug metabolite or drug host combinations, while also exposing the limitations of screening under uniform laboratory conditions. Although metabolomic analyses reveal extensive antibiotic-induced rewiring, their limited temporal and causal resolution restrict their ability to distinguish lethal processes from adaptive responses. Persistence presents a further challenge. Persister cells can arise through rapid, drug-induced transitions that are not necessarily linked to slow growth or classical toxin antitoxin systems. The mechanisms governing exit from persistence also represent an underexplored therapeutic opportunity.

Another major constraint is the integration of multiple stress responses within individual cells. Antibiotics commonly trigger overlapping DNA, envelope, translational, oxidative, and metabolic stresses, yet the rules by which these signals combine to produce death or survival remain obscure. Many stress signatures correlate with killing but lack predictive power, suggesting that amplifying individual stresses alone is unlikely to ensure lethality. Instead, identifying combinations of stresses that are intrinsically incompatible with recovery may provide a more robust strategy for drug development.

Finally, spatial structure and host context strongly reshape antibiotic action but are rarely captured during development. Biofilms generate steep gradients in metabolism, uptake, and stress responses, while host factors such as pH, oxygen availability, and immune activity can either potentiate or blunt drug effects. Early transport dynamics, including efflux, porin-mediated entry, and membrane energetics, further determine whether lethal intracellular concentrations are achieved. Together, these considerations argue for incorporating physiologically relevant and spatially structured models into antibiotic evaluation. Overall, advancing antibiotic development will require moving beyond target-centric optimisation toward strategies that account for metabolic state, physiological heterogeneity, stress integration, and host environment. Drugs or combinations that maintain efficacy across diverse contexts, limit rapid transitions into tolerance or persistence, and exploit context-specific vulnerabilities are likely to achieve greater clinical impact than agents optimised under idealised laboratory conditions.

## Figures and Tables

**Table 1:** Summary box of the major unresolved mechanistic questions in antibiotic-induced bacterial death.

Category	Conceptual Gaps	Addressed in Section(s)
1	Metabolic and physiological determinants of cell-death	Section 2, Section 5, Section 7 and Section 10
2	Stress responses and cell fate	Section 4 and Section 6
3	Persistence and phenotypic heterogeneity	Section 3 and Section 9
4	Spatial and structural contexts of antibiotic action	Section 8 and Section 11

## Data Availability

No new data were created or analyzed in this study. Data sharing is not applicable to this article.
